# Characterization of Microparticles after Hepatic Ischemia-Reperfusion Injury

**DOI:** 10.1371/journal.pone.0097945

**Published:** 2014-05-30

**Authors:** Christopher M. Freeman, Ralph C. Quillin, Gregory C. Wilson, Hiroyuki Nojima, Bobby L. Johnson, Jeffrey M. Sutton, Rebecca M. Schuster, John Blanchard, Michael J. Edwards, Charles C. Caldwell, Alex B. Lentsch

**Affiliations:** Department of Surgery, University of Cincinnati College of Medicine, Cincinnati, Ohio, United States of America; Universidade de Sao Paulo, Brazil

## Abstract

**Background:**

Hepatic ischemia-reperfusion (I/R) is a well-studied model of liver injury and has demonstrated a biphasic injury followed by recovery and regeneration. Microparticles (MPs) are a developing field of study and these small membrane bound vesicles have been shown to have effector function in other physiologic and pathologic states. This study was designed to quantify the levels of MPs from various cell origins–platelets, neutrophils, and endolethial cells–following hepatic ischemia-reperfusion injury.

**Methods:**

A murine model was used with mice undergoing 90 minutes of partial hepatic ischemia followed by various times of reperfusion. Following reperfusion, plasma samples were taken and MPs of various cell origins were labeled and levels were measured using flow cytometry. Additionally, cell specific MPs were further assessed by Annexin V, which stains for the presence of phosphatidylserine, a cell surface marker linked to apoptosis. Statistical analysis was performed using one-way analysis of variance with subsequent Student-Newman-Keuls test with data presented as the mean and standard error of the mean.

**Results:**

MPs from varying sources show an increase in circulating levels following hepatic I/R injury. However, the timing of the appearance of different MP subtypes differs for each cell type. Platelet and neutrophil-derived MP levels demonstrated an acute elevation following injury whereas endothelial-derived MP levels demonstrated a delayed elevation.

**Conclusion:**

This is the first study to characterize circulating levels of cell-specific MPs after hepatic I/R injury and suggests that MPs derived from platelets and neutrophils serve as markers of inflammatory injury and may be active participants in this process. In contrast, MPs derived from endothelial cells increase after the injury response during the reparative phase and may be important in angiogenesis that occurs in the regenerating liver.

## Introduction

Loss of functional liver mass can result from various mechanisms such as surgical resection, transplantation, traumatic injury, and acute liver failure. Following such an insult the liver has demonstrated regenerative potential, a pathway that is driven by a complex and integrated series of cell signaling resulting in cell growth, angiogenesis, tissue remodeling, etc. One such injury that has been investigated by our laboratory is hepatic ischemia-reperfusion (I/R) injury [1], [2], [3], [4]. This experimental injury model has demonstrated a biphasic response comprised of an acute injury phase resulting from oxidative stress and a later injury phase resulting from an intense inflammatory response that culminates in the hepatic recruitment of neutrophils and subsequent neutrophil-dependent parenchymal injury [5], [6], [7], [8], [9], [10]. Peak injury occurs within 24 hours of the initial insult. Hepatic recovery, repair, and regenerative responses after I/R are initiated 24 hours after injury, spans several days, and involves the clearance of dead tissue and the replacement of new functional liver mass by hepatocyte proliferation [5], [6].

During inflammatory processes such as I/R injury, cells in the vascular compartment, including platelets, endothelial cells, and neutrophils [11], have been shown to shed microparticles (MPs) [12]. These MP are small (0.3–1.0 µm), intact membrane bound vesicles that bleb off the cell membrane. MPs contain cytosolic components such as enzymes, transcription factors and RNA derived from the parental cell [13], [14], [15]. They are present during normal physiologic conditions [16] but have been demonstrated to increase during periods of stress. MPs may function as a means of intercellular communication by altering inflammation, increasing cell adherence, enhancing chemotaxis, inducing thrombosis, and effecting vascular function [17], [18], [19]. Additionally, MPs may play a role in angiogenesis and abrogation of the immune response. MPs express similar cell surface markers as their parent cells, facilitating quantification and characterization. Additionally, if derived from apoptotic cells they are characterized by the loss of plasma membrane polarity with phosphatidylserine being expressed on the outer leaflet of the lipid bilayer, as marked by Annexin V. In this study, we sought to characterize the relevant MP populations following hepatic I/R injury during the acute phase of injury and during the regenerative response.

## Materials and Methods

### Model of Hepatic I/R

Male C57bl6 (Jackson Laboratory, Bar Harbor, Maine, USA) weighing 22–28 g were used in these experiments. This project was approved by the University of Cincinnati Animal Care and Use Committee and was in compliance with the National Institutes of Health guidelines.

For hepatic I/R injury, mice underwent either sham surgery or I/R. Partial hepatic ischemia was induced as described previously [20]. Briefly, mice were anaesthetized with sodium pentobarbital (60 mg/kg subcutaneously). A midline laparotomy was performed and an atraumatic clip was used to interrupt blood supply to the left lateral and median lobes of the liver. The caudal lobes retained intact portal and arterial inflow and venous outflow, preventing intestinal venous congestion. After 90 minutes of partial hepatic ischemia, the clip was removed to initiate hepatic reperfusion. Sham control mice underwent the same protocol without vascular occlusion. Mice were sacrificed after the indicated periods of reperfusion, and blood samples were collected.

### Blood Samples, Microparticle Generation, and Analysis

**Figure 1:**
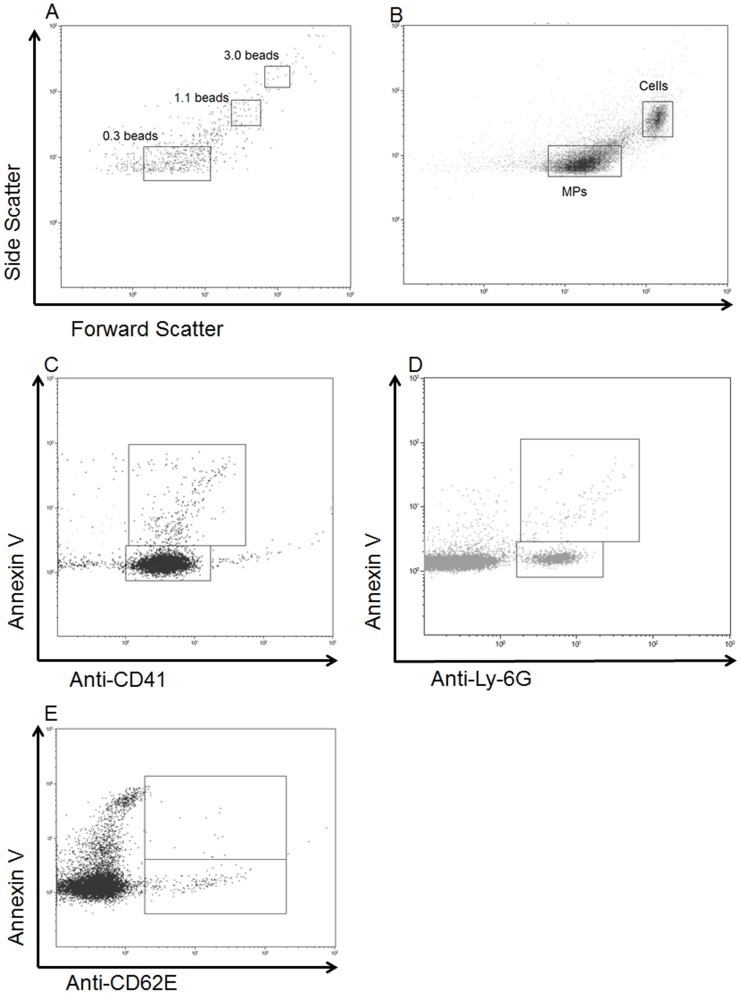
Characterization of microparticles following hepatic ischemia-reperfusion. Microparticles were isolated and analyzed as described in the methods. (A) Sizing for the microparticles was based on latex beads ranging in size from 0.5 to 3.0 µm. (B) Representative flow cytometric analysis of the forward and side scatter of microparticle mixture. (C) Representative flow cytometric analysis using Anti-CD41 (platelet specific cell marker) and Annexin V (apoptosis marker). (D) Representative flow cytometric analysis using Anti-Ly-6G (neutrophil specific marker) and Annexin V. (E) Representative flow cytometric analysis using Anti-CD62E (endothelial specific marker) and Annexin V.

Whole blood was obtained by cardiac puncture using heparin as an anticoagulant. Plasma was labeled for MPs of specific cell origin as previously described [21]. Forward and side-channels were set to analyze particles using 0.3, 1.1, and 3.0 µm latex beads for calibration (Figure 1A). The forward and side scatter gate was set based on size and vesicles within the 0.3–1.0 µm were identified as MPs (Figure 1B) and further analyzed. MPs were then assessed with both source specific markers and Annexin V (BD Pharmingen, San Diego, California, USA) which stains for the presence of phosphatidylserine, a cell surface marker linked to apoptosis [22]. Platelet-derived MP levels were assessed using the platelet-specific marker Anti-CD41 (clone: MWReg30, BD Pharmingen, San Diego, California, USA) (Figure 1C). Neutrophil-derived MP levels were assessed using neutrophil specific marker, Anti-Ly-6G (clone: 1A8, BD Pharmingen, San Diego, California, USA) [23] (Figure 1D). Endothelial-derived MP levels were assessed using endothelial cell specific marker Anti-CD-62E (clone 10E9.6, BD Pharmingen, San Diego, California, USA) (Figure 1E). Flow cytometry data acquisition and analysis were performed using a Beckman Coulter Epic flow cytometer with Kaluza software (Indianapolis, IN, USA).

### Statistical Analysis

All data are expressed as mean ± standard error of the mean (SEM). Data were analyzed with a one-way analysis of variance with subsequent Student-Newman-Keuls test. Differences were considered significant when *P*<0.05. All statistical analysis was performed using SigmaPlot, Version 11 (Systat Software, Inc. San Jose, California, USA).

## Results

### Platelet-derived MPs are Increased Immediately after Hepatic Reperfusion

**Figure 2:**
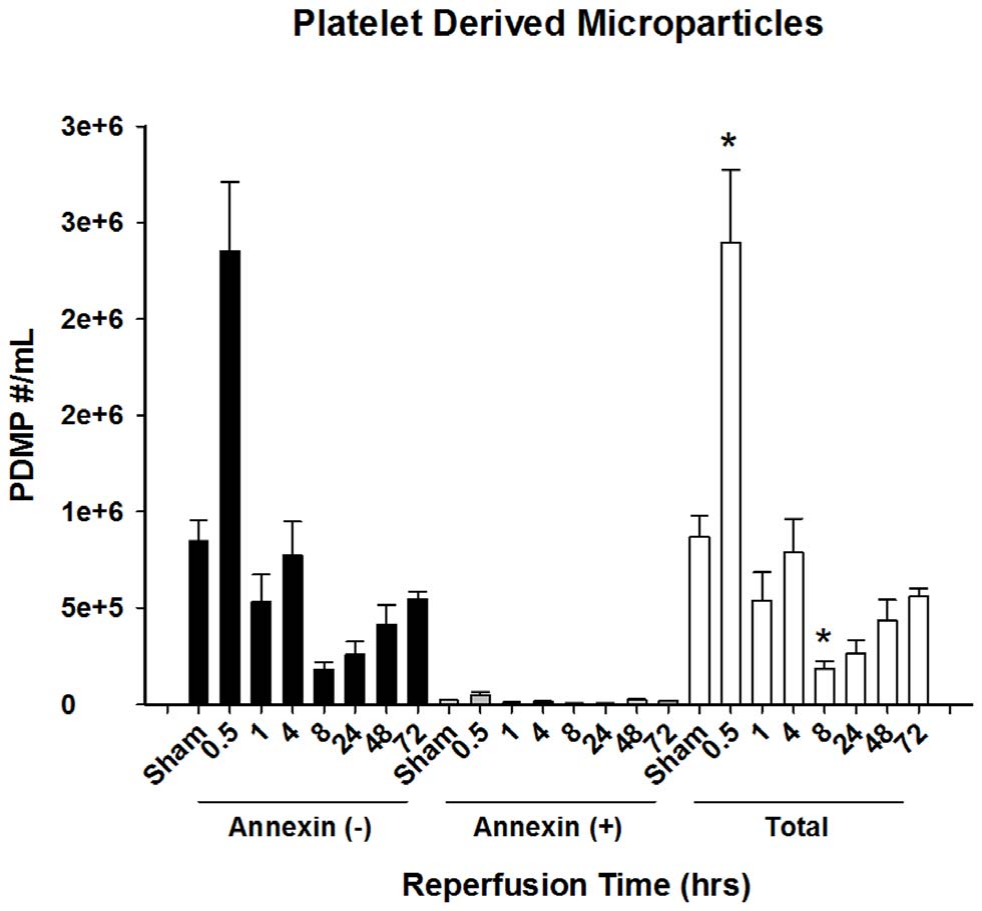
Characterization of platelet-derived microparticles after hepatic ischemia-reperfusion injury. Platelet-derived microparticles present in plasma in varying times of reperfusion following hepatic ischemic injury. Microparticle levels are subdivided as total MP, Annexin negative, and Annexin positive MPs. Data presented are mean ± standard error of mean. N = 5–20 for all groups. *P<0.05 compared to sham.

We found that platelet-derived MPs were significantly increased within 30 minutes of hepatic reperfusion (Figure 2). Interestingly, within 1 hour of reperfusion, the number of circulating platelet-derived MPs returned to sham levels. Eight hours after reperfusion, the number of circulating platelet-derived MPs was significantly lower than the sham control group, but returned to sham levels within 48 hours of reperfusion. Analysis of Annexin V labeling showed that all platelet-derived MPs were negative (Figure 2), which is consistent with the fact that platelets do not undergo apoptosis.

### Neutrophil-derived MPs are Increased during the Acute Inflammatory Response Induced by I/R

**Figure 3:**
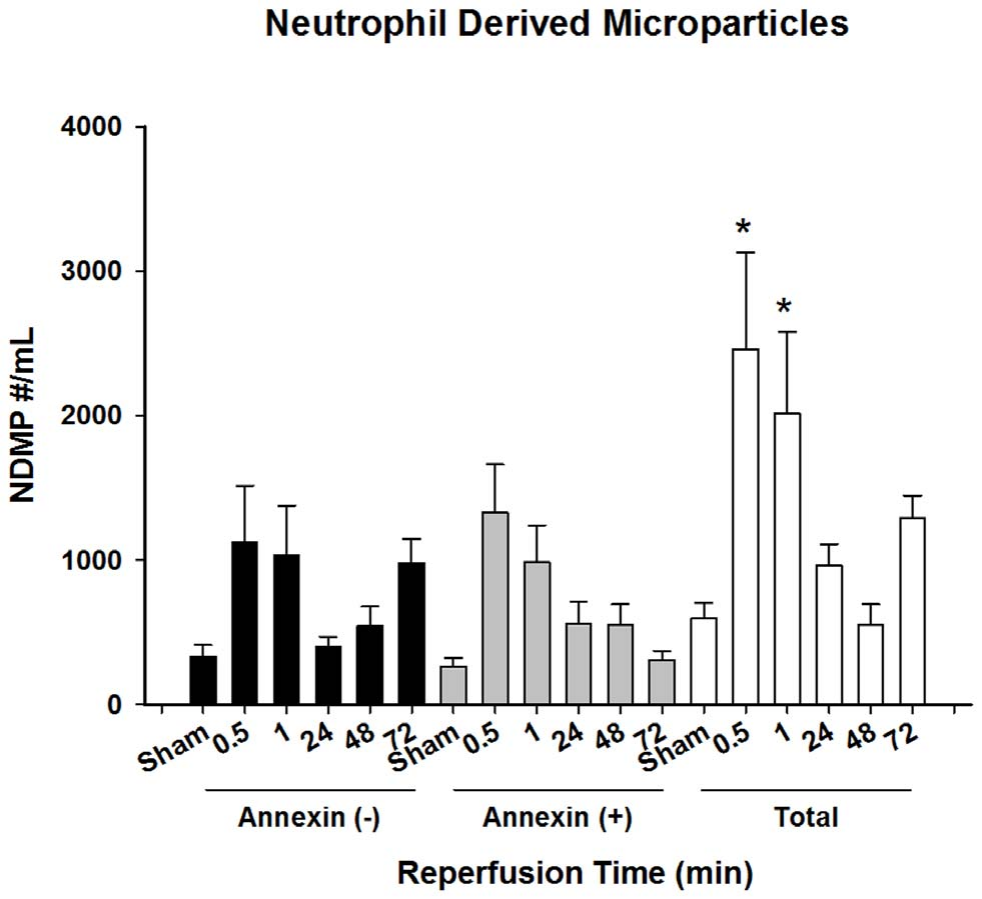
Characterization of neutrophil-derived microparticles after hepatic ischemia-reperfusion injury. Neutrophil-derived microparticles present in plasma in varying times of reperfusion following hepatic ischemic injury. Microparticle levels are subdivided as total MP, Annexin negative, and Annexin positive MPs. Data presented are mean ± standard error of mean. N = 5–15 for all groups. *P<0.05 compared to sham.

Similar to platelet-derived MPs, the number of circulating neutrophil-derived MPs was significantly increased within 30 minutes of reperfusion (Figure 3). However, levels of neutrophil-derived MPs remained elevated during the acute inflammatory phase of I/R injury and returned to baseline levels by 24 hours after reperfusion. Analysis of Annexin V staining demonstrated that neutrophil-derived MPs were comprised of two distinct poplulations. Those MPs originating from activated cells (Annexin V negative) showed an initial peak at 30 minutes after reperfusion, but returned to sham levels by 24 hours. However, levels at subsequent time points increased, and by 72 hours after reperfusion, the number of these MPs had approximated levels at the 0.5 and 1 hour time points. In contrast, those MPs originating from apoptotic cells (Annexin V positive) peaked at 30 minutes post-injury and trended downward over all subsequent time points.

### Endothelial Cell-derived MPs Increase during the Recovery/Regenerative Phase

**Figure 4:**
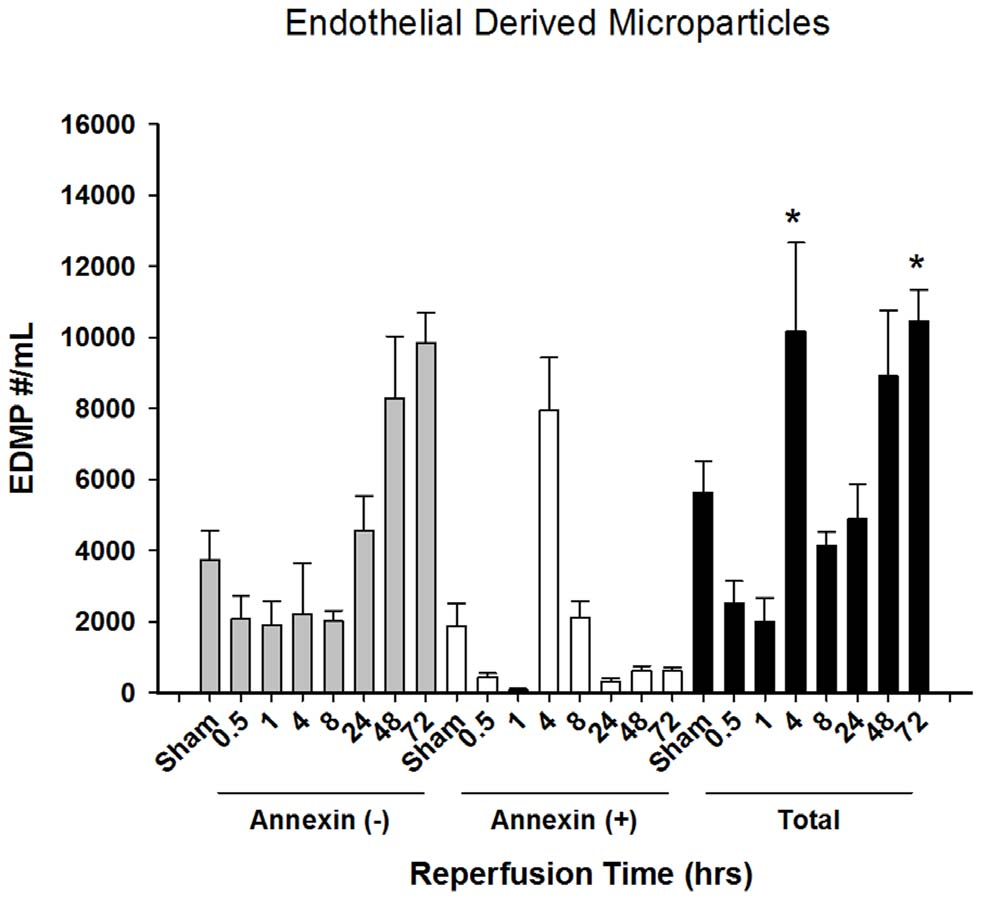
Characterization of endothelial cell-derived microparticles after hepatic ischemia-reperfusion injury. Endothelial cell-derived microparticles present in plasma in varying times of reperfusion following hepatic ischemic injury. Microparticle levels are subdivided as total MP, Annexin negative, and Annexin positive MPs. Data presented are mean ± standard error of mean. N = 6–20 for all groups. *P<0.05 compared to sham.

Unlike platelet- and neutrophil-derived MPs, endothelial cell-derived MPs did not increase acutely after reperfusion (Figure 4). Endothelial cell-derived MPs increased significantly 4 hours after reperfusion, but were back to sham levels by 8 hours. However, sustained increases were observed at 48 and 72 hours after reperfusion, at a time when the liver is actively undergoing repair and regeneration. Analysis of Annexin V staining was very interesting, with the vast majority of MPs at the 4-hour time point staining positive, indicating they originated from apoptotic cells (Figure 4). In contrast, almost all of the MPs at the 48 and 72-hour time points originated from activated cells (Annexin V negative).

## Discussion

In this study, we observed that MPs from varying sources show an increase in circulating levels following hepatic I/R injury. These findings are supported by the literature, which has demonstrated increased circulating levels of MPs following inflammation, tissue necrosis, and vascular injury [21], [24], [25]. However, the timing of the appearance of different MP subtypes, and perhaps their particular role in the response to I/R injury, appears to differ for each cell type. Based on our results, we believe that MPs may contribute to both the acute inflammatory injury as well as the reparative and regenerative responses after I/R injury.

Following I/R injury, the majority (approximately 80%) of circulating MPs were derived from platelets. This finding is consistent with the literature in septic patients [24], [26]. Platelet-derived MPs peaked within 30 minutes of reperfusion, suggesting they may likely contribute to the early injury phase. Platelet-derived MPs have been shown to directly activate both neutrophils and macrophages, facilitating their recruitment to sites of inflammation by up regulating adhesion molecules on endothelial cells [27], [28], [29], [30], [31]. These very same effects occur in the context of hepatic I/R injury, in addition to endothelial cell injury, for which platelets have been previously attributed [32], [33].

Similar to platelet-derived MPs, the levels of circulating neutrophil-derived MPs were also increased acutely after reperfusion. However, they remained elevated during the acute period of inflammation, suggesting they may play an active role in the inflammation and injury that occurs after I/R. This concept is supported by other studies that demonstrate that neutrophil-derived MPs promote coagulation, thrombosis, and complement activation, adhere to and activate endothelial cells [34] and can stimulate endothelial cells to release inflammatory cytokines like IL-6 and IL-8 [35], [36]. Interestingly, neutrophil-derived MPs appeared to originate from both activated and apoptotic cells. MPs originating from apoptotic cells peaked at 30 minutes post-injury and subsequently decreased in number, whereas MPs from activated cells demonstrated showed an acute increase, followed by a return to baseline levels and then a second increase during the recovery phase (72 hours). However, the significance of these differences is unclear, since the total number of neutrophil-derived MPs was not significantly elevated after 1 hour of reperfusion. However, significant neutrophil accumulation does not occur at these early time points, suggesting that these MPs may be derived from circulating neutrophils that are activated by soluble mediators released from the post-ischemic liver [7], [8].

An altogether different phenotype was found for endothelial cell-derived MPs. Unlike MPs derived from platelets and neutrophils, endothelial cell-derived MPs did not increase acutely after reperfusion. An increase was observed 4 hours after reperfusion, but these were almost entirely derived of Annexin V positive MPs, suggesting that this increase simply reflects an increase in endothelial cell injury, which is known to occur after reperfusion. This is consistent with previous findings that endothelial cell-derived MPs serve as markers of endothelial cell dysfunction [37], [38]. More interesting are the increases in endothelial cell-derived MPs that were observed 48 and 72 hours after reperfusion. At these time points, the liver is actively repairing and regenerating. It is highly likely that these MPs are associated with revascularization of the regenerating liver. In fact, previous studies have implicated endothelial cell-derived MPs as mediators of angiogenesis [39], [40].

While our observations are novel in that they characterize different types of MPs generated as a result of hepatic I/R injury, it is difficult to ascribe direct functions to these different MPs since they cannot be selectively blocked or neutralized. As such, we are resigned to extrapolate the functions that have been described for these MPs in other systems [19], [21], [26], [27], [28], [30], [36] until we are able to isolate enough of the different MP subsets to administer exogenously during the injury process.

In summary, our study is the first to characterize circulating levels of cell-specific MPs after hepatic I/R injury. Our data suggest that MPs derived from platelets and neutrophils serve as markers of inflammatory injury and may be active participants in this process. In contrast, we found that MPs derived from activated endothelial cells increase after the injury response during the reparative phase and may be important in angiogenesis that occurs in the regenerating liver. Additional investigation into the precise roles of each of these MP subsets is warranted to determine their biological functions in liver biology and pathology.
